# Assessment of Academic Achievement of Female Physicians in Japan

**DOI:** 10.1001/jamanetworkopen.2020.9957

**Published:** 2020-07-09

**Authors:** Kaori Kono, Takashi Watari, Yasuharu Tokuda

**Affiliations:** 1 Faculty of Medicine, Shimane University, Shimane, Japan; 2 Postgraduate Clinical Training Center, Shimane University Hospital, Shimane, Japan; 3 Okinawa Muribushi Project for Teaching Hospitals, Okinawa, Japan

## Abstract

This survey study examines trends in the gender distribution of academic positions in medical schools and university-affiliated hospitals in Japan from 1980 to 2018.

## Introduction

Despite the global increase in the number of female physicians, there are still fewer women than men in key positions.^1^ In a US-based study,^2^ salaries of female physicians were significantly lower than those of male physicians, although there was no gender difference in income among nonphysician health care workers. Women who have worked longer face greater pay discrimination.^2^ A barrier to academic and financial advancement—known as the glass ceiling—still prevails globally for female physicians, and Japan is no exception.

## Methods

Because we used only Japanese National Basic School Survey data that had already been published by the Ministry of Education, Culture, Sports, Science and Technology, this study did not require institutional review board approval or informed consent. The institutional review board of Shimane University does not consider this type of study human research and therefore does not require its review.This was a survey study and conducted in accordance with American Association for Public Opinion Research guidelines. We analyzed Japanese government statistical data to elucidate trends in the gender distribution of university academic positions, including professors, associate professors, lecturers, and assistant professors, in medical schools and university-affiliated hospitals from 1980 to 2018. We also obtained statistical data from the Ministry of Health, Labour, and Welfare to trace the changes in the rate of female physicians registered with the Ministry from 1980 to 2016. Data analysis was performed in December 2019 with Excel for Mac statistical software version 16.16.22 (Microsoft Corp).

## Results

Our findings show that, although the proportion of female physicians in all 4 academic positions has increased over the 4 decades from 10.0% to 21.1% (Figure), the proportion of female professors was still less than 2% from 1980 to 1992; this proportion has remained stagnant since 2004, at less than 10%. Compared to the assistant professor position, the associate professor and lecturer positions had noticeably lower proportions of women in 2018, at 13.1% and 16.8%, respectively. Conversely, the proportion of female assistant professors has consistently increased since 1995. It should be noted that this proportion overtook the proportion of female physicians in 1994, eventually reaching 29.7% in 2018 compared with 21.1% for female physicians.

**Figure.**
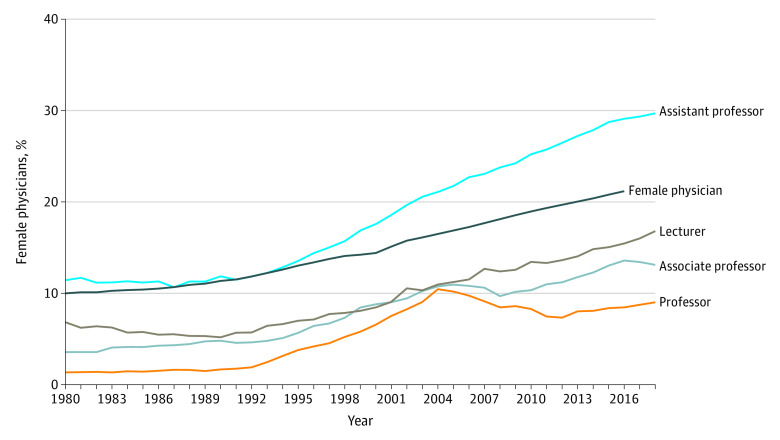
Trends in the Proportion of Academic Positions for Female Physicians, Japan, 1980-2018

## Discussion

The study had some limitations. We lacked detailed information about some variables, such as the number of new female professors; however, our national data were highly comprehensive and suggest that the promotion of female physicians in Japanese academic medicine is stifled (ie, the glass ceiling). Japan has aligned its gender policies with the international community in terms of the mainstreaming of gender issues and has implemented the Equal Employment Opportunity Law in 1986 and the Basic Act of Gender Equal Society in 1999. These laws may have led to an increase in the proportion of female physicians and faculty members. However, there has not been much progress toward eliminating gender disparity. According to a 2020 report,^3^ Japan’s Global Gender Index was the second lowest among Organisation for Economic Co-operation and Development countries and the worst among G7 countries. Moreover, a 2018 study^4^ reported that several Japanese medical schools continued to systematically discriminate against female applicants in the entrance examination by unfairly reducing test scores by as much as 20%. Thus, recent data suggest that Japanese medical schools continue to maintain a gender bias that goes against basic ethics and human rights.

To overcome these problems, we believe the following initiatives are important and should be implemented: (1) establishment of a program to support women’s advancement in academia (eg, by providing in-hospital childcare centers or exempting women from night shift and overtime work); (2) sponsorships for strong mentors to act as role models for junior women on faculties that traditionally offer fewer opportunities for women because of shorter working hours^5^; (3) promotion of leadership and research skills among female faculty members^6^; and (4) establishment of proportion targets for female physicians among faculty members in Japanese medical schools. These should be monitored to ensure accountability in achieving these initiative goals and to protect basic ethical principles.
